# Association of Sex and Age With Mild Traumatic Brain Injury–Related Symptoms: A TRACK-TBI Study

**DOI:** 10.1001/jamanetworkopen.2021.3046

**Published:** 2021-04-06

**Authors:** Harvey S. Levin, Nancy R. Temkin, Jason Barber, Lindsay D. Nelson, Claudia Robertson, Jeffrey Brennan, Murray B. Stein, John K. Yue, Joseph T. Giacino, Michael A. McCrea, Ramon Diaz-Arrastia, Pratik Mukherjee, David O. Okonkwo, Kim Boase, Amy J. Markowitz, Yelena Bodien, Sabrina Taylor, Mary J. Vassar, Geoffrey T. Manley, and the TRACK-TBI Investigators, Opeolu Adeoye, Neeraj Badjatia, M. Ross Bullock, Randall Chesnut, John D. Corrigan, Karen Crawford, Sureyya Dikmen, Ann-Christine Duhaime, Richard Ellenbogen, V. Ramana Feeser, Adam R. Ferguson, Brandon Foreman, Raquel Gardner, Etienne Gaudette, Luis Gonzalez, Shankar Gopinath, Rao Gullapalli, J. Claude Hemphill, Gillian Hotz, Sonia Jain, C. Dirk Keene, Frederick K. Korley, Joel Kramer, Natalie Kreitzer, Chris Lindsell, Joan Machamer, Christopher Madden, Alastair Martin, Thomas McAllister, Randall Merchant, Amber Nolan, Laura B. Ngwenya, Florence Noel, Eva Palacios, Ava Puccio, Miri Rabinowitz, Jonathan Rosand, Angelle Sander, Gabriella Satris, David Schnyer, Seth Seabury, Xiaoying Sun, Arthur Toga, Alex Valadka, Kevin Wang, Esther Yuh, Ross Zafonte

**Affiliations:** 1 Baylor College of Medicine, Houston, Texas; 2 Michael E. DeBakey Veterans Affairs Medical Center, Houston, Texas; 3 Department of Neurological Surgery, University of Washington, Seattle; 4 Department of Biostatistics, University of Washington, Seattle; 5 Department of Neurosurgery, Medical College of Wisconsin, Milwaukee; 6 Department of Neurology, Medical College of Wisconsin, Milwaukee; 7 University of California, San Diego; 8 University of California, San Francisco; 9 Spaulding Rehabilitation Center, Boston, Massachusetts; 10 Massachusetts General Hospital, Boston; 11 Department of Neurology, University of Pennsylvania, Philadelphia; 12 Department of Neurosurgery, University of Pittsburgh, Pittsburgh; 13 University of Cincinnati, Ohio; 14 University of Maryland, College Park; 15 University of Miami, Miami, Florida; 16 Ohio State University, Columbus; 17 University of Southern California, Los Angeles, California; 18 Department of Rehabilitation Medicine, University of Washington, Seattle; 19 MassGeneral Hospital for Children, Boston, Massachusetts; 20 Virginia Commonwealth University, Richmond, Virginia; 21 TIRR Memorial Hermann, Houston, Texas; 22 University of Michigan, Ann Arbor; 23 Vanderbilt University, Nashville, Tennessee; 24 University of Texas Southwestern Medical School, Dallas; 25 Indiana University, Bloomington; 26 University of Texas at Austin, Austin; 27 University of Florida, Gainesville; 28 Harvard Medical School, Boston, Massachusetts

## Abstract

**Importance:**

Knowledge of differences in mild traumatic brain injury (mTBI) recovery by sex and age may inform individualized treatment of these patients.

**Objective:**

To identify sex-related differences in symptom recovery from mTBI; secondarily, to explore age differences within women, who demonstrate poorer outcomes after TBI.

**Design, Setting, and Participants:**

The prospective cohort study Transforming Research and Clinical Knowledge in Traumatic Brain Injury (TRACK-TBI) recruited 2000 patients with mTBI from February 26, 2014, to July 3, 2018, and 299 patients with orthopedic trauma (who served as controls) from January 26, 2016, to July 27, 2018. Patients were recruited from 18 level I trauma centers and followed up for 12 months. Data were analyzed from August 19, 2020, to March 3, 2021.

**Exposures:**

Patients with mTBI (defined by a Glasgow Coma Scale score of 13-15) triaged to head computed tomography in 24 hours or less; patients with orthopedic trauma served as controls.

**Main Outcomes and Measures:**

Measured outcomes included (1) the Rivermead Post Concussion Symptoms Questionnaire (RPQ), a 16-item self-report scale that assesses postconcussion symptom severity over the past 7 days relative to preinjury; (2) the Posttraumatic Stress Disorder Checklist for the *Diagnostic and Statistical Manual of Mental Disorders* (Fifth Edition) (PCL-5), a 20-item test that measures the severity of posttraumatic stress disorder symptoms; (3) the Patient Health Questionnaire-9 (PHQ-9), a 9-item scale that measures depression based on symptom frequency over the past 2 weeks; and (4) the Brief Symptom Inventory-18 (BSI-18), an 18-item scale of psychological distress (split into Depression and Anxiety subscales).

**Results:**

A total of 2000 patients with mTBI (1331 men [67%; mean (SD) age, 41.0 (17.3) years; 1026 White (78%)] and 669 women [33%; mean (SD) age, 43.0 (18.5) years; 505 (76%) White]). After adjustment of multiple comparisons, significant TBI × sex interactions were observed for cognitive symptoms (B = 0.76; 5% false discovery rate–corrected *P* = .02) and somatic RPQ symptoms (B = 0.80; 5% false discovery rate–corrected *P* = .02), with worse symptoms in women with mTBI than men, but no sex difference in symptoms in control patients with orthopedic trauma. Within the female patients evaluated, there was a significant TBI × age interaction for somatic RPQ symptoms, which were worse in female patients with mTBI aged 35 to 49 years compared with those aged 17 to 34 years (B = 1.65; *P* = .02) or older than 50 years (B = 1.66; *P* = .02).

**Conclusions and Relevance:**

This study found that women were more vulnerable than men to persistent mTBI-related cognitive and somatic symptoms, whereas no sex difference in symptom burden was seen after orthopedic injury. Postconcussion symptoms were also worse in women aged 35 to 49 years than in younger and older women, but further investigation is needed to corroborate these findings and to identify the mechanisms involved. Results suggest that individualized clinical management of mTBI should consider sex and age, as some women are especially predisposed to chronic postconcussion symptoms even 12 months after injury.

## Key Points

**Question:**

Do postacute mild traumatic brain injury (mTBI) symptoms differ between men and women?

**Findings:**

In this cohort study of 2000 patients with mTBI, the severity of cognitive and somatic symptoms on the Rivermead Post Concussion Symptoms Questionnaire at 12 months after injury was significantly worse in women than in men, whereas this difference was not significant in an orthopedic trauma control group. The association between mTBI and somatic symptoms was greater in women aged 35 to 49 years than those aged 17 to 34 years or older than 50 years.

**Meaning:**

Sex and age may be important factors in the individualized, postacute treatment of patients with mTBI.

## Teaser

This cohort study uses data from the Transforming Research and Clinical Knowledge in Traumatic Brain Injury (TRACK-TBI) study to assess whether patient sex and age are associated with mild traumatic brain injury symptoms.

## Introduction

Nearly 3 million cases of traumatic brain injury (TBI) are treated annually in the US, with mild TBI (mTBI) accounting for almost 85% of this population.^1^ Sex differences in recovery from TBI have recently attracted research and clinical attention.^2,3^ For example, 60% of studies concerning recovery from mTBI of all causes report that women experience slower resolution of postconcussion symptoms (PCS), more severe emotional symptoms, and worse outcomes than male patients.^2^ Similarly, consensus guidelines for clinical management of sports-related concussion include female sex as a risk factor for persistent PCS and delayed return to play.^4^

Despite progress in characterizing sex differences in the trajectory of recovery from mTBI, there remain gaps in knowledge of the epidemiology, mechanisms, and effect of persisting PCS in women. Diverse factors could explain sex differences in mTBI symptoms and recovery, including hormonal contributions,^5,6^ sex differences in premorbid risk factors for prolonged mTBI recovery (eg, psychiatric disorders),^7^ differences in psychosocial stress,^5,8^ and differences in general willingness or propensity to report symptoms.^3^ To advance toward a better understanding of the mechanisms of sex differences in recovery, basic questions about the phenomenon should be addressed, such as whether sex differences in recovery are unique to mTBI (vs generalize to nonbrain injury, which might implicate nonspecific contributions, such as sex differences in reporting biases), and by looking at the contribution of age to the poorer outcomes experienced by women (which might be a proxy for circulating hormones or reflect differences between age cohorts in psychosocial stress).

First, it is unclear whether sex differences in recovery also occur in patients with traumatic injuries not involving the brain. Second, potential interactions of sex with age have not been studied extensively.^5,6,8^ There are several reasons women at certain ages may be especially vulnerable to the consequences of mTBI. For example, circulating levels of sex hormones, which decrease in women from about 35 to 50 years of age, may moderate recovery, but the evidence for a causal linkage is preliminary.^6^ Preclinical research has established that estrogen is neuroprotective, and progesterone is an effective treatment of acute TBI, but these findings have not been corroborated in clinical studies.^9,10^

It is also plausible that sex differences in vulnerability to posttraumatic stress disorder (PTSD) and depression may contribute to the observed sex differences in recovery from mTBI.^7,11^ Similarly, more severe psychosocial stress^5,8,11^ and greater proclivity for self-disclosure of symptoms^3^ have been cited as drivers of more persistent PCS and emotional disturbance after mTBI in women than men. These studies have suggested that women resuming child care, domestic chores, and external jobs may experience greater psychosocial stress than younger and older women.^5,8^

The Transforming Research and Clinical Knowledge in Traumatic Brain Injury (TRACK-TBI) pilot study, a forerunner to the present project, reported a sex × age interaction on mTBI symptom measures at 6 months after injury in 100 young adult patients with mTBI aged 18 to 39 years, with more persistent TBI-related symptoms in women aged 30 to 39 years than younger women and men in both the 19- to 29-year and 30- to 39-year subgroups. However, it was unclear whether sex differences on symptom measures were specific to mTBI, because the TRACK-TBI study lacked a non-TBI traumatic injury control group.

The primary aim of the present study was to characterize sex differences in the symptom-based recovery measures in the prospective, multicenter TRACK-TBI study. Patients with mTBI or orthopedic trauma completed symptom inventories longitudinally over 12 months after injury. We hypothesized that PCS, symptoms of PTSD, depression, and anxiety would be more severe and persistent in women than in men with mTBI. In addition, we hypothesized that the sex difference in injury-related symptoms would be greater in patients with mTBI than in a control group that sustained orthopedic injuries. The rationale for this comparison is that subgroups of PCS are nonspecific (eg, anxiety, irritability, sleep disturbance, fatigue, depression, frustration, and restlessness) and may arise after traumatic injury to body regions other than the head and brain. As a secondary objective, we also explored age differences within female patients with mTBI. Age strata of 18 to 34 years, 35 to 49 years, and older than 50 years were selected based on epidemiologic data suggesting that circulating female sex hormones decline around age 35 years up to the point of menopause (median age, 51 years).^12,13,14^ We hypothesized that, if female sex hormones are a primary driver of sex differences in mTBI recovery, then we may observe a monotonic relationship between age and symptoms. However, given prior findings that women in late early or early middle adulthood may be at highest risk of persisting PCS,^5^ perhaps owing to psychosocial factors or a combination of psychosocial factors and hormonal changes, we also considered the possibility that women aged 35 to 49 years may demonstrate the most severe symptoms after mTBI.

## Methods

### Patients and Study Design

This cohort study included 2000 patients with mTBI aged 17 years to older than 90 years (1331 men and 669 women) who were recruited at any of the 18 level I trauma center sites that were part of the TRACK-TBI study. A total of 299 similarly aged patients with orthopedic trauma injury served as controls (OTCs; 199 men, 100 women) and were recruited at the same sites. The patients with mTBI were enrolled between February 26, 2014, and July 3, 2018, and the OTCs were enrolled between January 26, 2016, and July 27, 2018. A participant flow diagram indicating selection of the present sample of patients with mTBI from the total TRACK-TBI cohort is shown in eFigure 1 in the Supplement. For each end point, the numbers of patients who withdrew, died, or were lost to follow-up, as well as the number in each group who provided data, are shown (eFigure 1 in the Supplement). Data collection included demographic features, medical and psychiatric history, clinical injury variables, and neuropsychological assessments using the TBI Common Data Elements.^15^ Outcome assessments were performed at 2 weeks, 3 months (by telephone), 6 months, and 12 months.

The study was approved by the institutional review board of each enrolling institution and was led by the University of California, San Francisco. Patients or their legally authorized representatives provided written informed consent and received financial compensation. This report was completed in accordance with the Strengthening the Reporting of Observational Studies in Epidemiology (STROBE) reporting guideline.

### Eligibility Criteria

Inclusion criteria for the mTBI group were (1) presenting to a recruitment center within 24 hours after injury; (2) a clinical computed tomography (CT) scan within 24 hours after injury; (3) exhibiting or reporting alteration of consciousness or amnesia; and (4) Glasgow Coma Scale (GCS) score in the emergency department of 13 to 15 points. Control patients with orthopedic trauma presented at the same emergency departments with mild orthopedic injuries without evidence of trauma to the head, no report of alteration of consciousness, and no period of amnesia. Exclusion criteria in both groups included being in police custody, pregnancy, sustaining a nonsurvivable injury, the existence of a preinjury debilitating psychiatric disorder or neurologic disease, and non-English speaking with the exception of Spanish speakers at certain centers.

### Outcome Measures

#### Rivermead Post Concussion Symptoms Questionnaire

The Rivermead Post Concussion Symptoms Questionnaire (RPQ)^16^ consists of 16 items in which patients rate each symptom (0-4) for severity (relative to preinjury) over the past 7 days; symptoms that did not differ in severity from preinjury were scored as 0. Total scores ranged from 0 to 64 with higher scores indicating more severe symptoms. Based on a previous factor analysis,^17^ the RPQ is divided into 3 domains: somatic (eg, headaches, dizziness, fatigue), emotional (eg, irritability, depression, frustration), and cognitive (eg, poor concentration, forgetfulness). These are the primary outcomes for this paper.

#### Posttraumatic Stress Disorder Checklist–5

The Posttraumatic Stress Disorder Checklist^18^ for the *Diagnostic and Statistical Manual of Mental Disorders* (Fifth Edition) (PCL-5) is a self-report scale for past exposure to a very stressful experience, such as a natural disaster or crime, that threatened death or serious injury to the participant, was witnessed by the participant, or which they heard about. Using this worst event experienced as a reference, the participant was then asked to rate the degree to which they were bothered by problems related to the event over the past month. The scale has 20 items, which the participant rated on a 0 to 4 scale (0, not at all bothered, to 4, extremely bothered); the total score ranges from 0 to 80.

#### Patient Health Questionnaire-9 and Brief Symptom Inventory-18

The Patient Health Questionnaire (PHQ-9)^19^ is a self-report scale of 9 depressive symptoms that asks the respondent to report the proportion of days over the past 2 weeks that the symptom was experienced, from 0, not at all, to 3, nearly every day. The total score ranges from 0 to 27.

The Brief Symptom Inventory-18 (BSI-18)^20^ is an 18-item scale of psychological distress that was also administered. The subscales of depression and anxiety were analyzed. The range of scores is 36 to 80.

### Statistical Analysis

Differences in subject characteristics were assessed for statistical imbalance using Mann-Whitney U tests (continuous and ordinal variables) and Fisher exact tests (categorical variables). Demographic variables (Table 1), including race/ethnicity, were coded in accordance with the National Institutes of Neurological Disorders and Stroke TBI Common Data Elements and were self-reported by participants or their legally authorized representatives.

**Table 1.** Preinjury Demographics, Clinical History, and TBI Variables^a^

Characteristic	mTBI, No. (%)		*P* value	Trauma controls, No. (%)		*P* value
	Men	Women		Men	Women	
No. (%)	1331 (67)	669 (33)	NA	199 (67)	100 (33)	NA
Age, mean (SD), y	41.0 (17.3)	43.0 (18.5)	.07	38.9 (14.6)	43.3 (16.6)	.03
Age, y						
17-34	593 (45)	273 (41)	.02	92 (46)	39 (39)	.04
35-49	311 (23)	138 (21)		56 (28)	21 (21)	
≥50	427 (32)	258 (39)		51 (26)	40 (40)	
Race/ethnicity						
White	1026 (78)	505 (76)	.82	156 (80)	75 (76)	.82
Black	211 (16)	119 (18)		28 (14)	18 (18)	
Asian	46 (4)	22 (3)		5 (3)	3 (3)	
Mixed race	22 (2)	12 (2)		2 (1)	2 (2)	
Other^b^	9 (1)	3 (0)		3 (2)	1 (1)	
Unknown	17	8	NA	5	1	NA
Hispanic	NA	NA	NA	NA	NA	NA
No	1031 (78)	538 (81)	.14	142 (73)	79 (80)	.20
Yes	284 (22)	124 (19)		53 (27)	20 (20)	
Unknown^c^	16	7	NA	4	1	NA
Education						
Mean (SD), y	13.4 (2.9)	13.7 (2.9)	.001	13.3 (3.1)	14.7 (2.4)	<.001
Unknown^c^	75	27	NA	5	2	NA
Insurance						
Insured/Medicare	803 (64)	426 (67)	<.001	116 (62)	68 (72)	.03
Medicaid/other	155 (12)	118 (18)		24 (13)	17 (18)	
Uninsured	296 (24)	94 (15)		47 (25)	10 (11)	
Unknown^c^	77	31	NA	12	5	NA
Living situation						
Independent living	1011 (80)	562 (87)	.002	152 (80)	89 (92)	.002
Living with other	235 (19)	82 (13)		38 (20)	6 (6)	
Other	11 (1)	4 (1)		1 (1)	2 (2)	
Unknown^c^	74	21	NA	8	3	NA
Previous TBI						
No	923 (76)	506 (83)	.001	152 (83)	78 (86)	.57
ED visit	170 (14)	73 (12)		20 (11)	6 (7)	
Hospital admit	120 (10)	34 (6)		12 (7)	7 (8)	
Unknown^c^	118	56	NA	15	9	NA
Drug use history						
None	824 (66)	495 (78)	<.001	122 (64)	74 (77)	.02
Yes, no trouble	372 (30)	130 (20)		60 (31)	20 (21)	
Yes, with trouble	61 (5)	12 (2)		9 (5)	2 (2)	
Unknown^c^	74	32	NA	8	4	NA
Psychiatric history						
None	940 (71)	378 (57)	<.001	136 (69)	50 (50)	.001
Received help (no regular meds)	201 (15)	127 (19)		34 (17)	20 (20)	
Used meds regularly (no hosp)	149 (11)	136 (20)		23 (12)	24 (24)	
Hospitalized	39 (3)	28 (4)	NA	5 (3)	6 (6)	NA
Unknown^c^	2	0	NA	1	0	NA
Psychiatric disorder						
Anxiety	113 (9)	130 (19)	<.001	24 (12)	19 (19)	.12
Depression	134 (10)	151 (23)	<.001	20 (10)	26 (26)	.001
Sleep disorders	39 (3)	34 (5)	.02	3 (2)	3 (3)	.41
Bipolar disorder	14 (1)	15 (2)	.05	0	1 (1)	.34
Schizophrenia	3 (0)	2 (0)	1.00	0	1 (1)	.34
PTSD	19 (1)	15 (2)	.20	3 (2)	0	.55
Other	33 (2)	17 (3)	1.00	7 (4)	2 (2)	.72
Unknown^c^	2	0	NA	1	0	NA
Injury cause						
Road traffic accident	729 (55)	410 (62)	.001	72 (38)	35 (36)	<.001
Fall	361 (27)	184 (28)		53 (28)	50 (52)	
Other accident	81 (6)	27 (4)		36 (19)	8 (8)	
Violence	110 (8)	18 (3)		3 (2)	0	
Other	46 (3)	27 (4)		27 (14)	4 (4)	
Unknown^c^	4	3	NA	8	3	NA
ED GCS, mean (SD)	14.7 (0.6)	14.7 (0.5)	.10	NA	NA	NA
CT, positive						
No	781 (61)	438 (67)	.004	NA	NA	NA
Yes	506 (39)	211 (33)				
Unknown^c^	44	20	NA			
LOC duration						
None	112 (12)	74 (16)	.006	NA	NA	NA
1-29 min	719 (80)	360 (79)				
30 min-24 h	63 (7)	20 (4)				
>24 h	6 (1)	0				
Unknown^c^	431	215	NA			
PTA duration						
None	173 (19)	92 (20)	.15	NA	NA	NA
1-29 min	376 (41)	203 (44)				
30 min-24 h	312 (34)	134 (29)				
>24 h	58 (6)	28 (6)				
Unknown^c^	412	212	NA			
Highest level of care						
ED	325 (24)	193 (29)	.001	64 (32)	44 (44)	.13
Ward	556 (42)	296 (44)		119 (60)	46 (46)	
ICU	450 (34)	180 (27)		16 (8)	10 (10)	
Litigation at 12 mos						
No	665 (81)	347 (75)	.04	97 (83)	60 (85)	.84
Yes	161 (19)	113 (25)		20 (17)	11 (15)	
Unknown^c^	505	209	NA	82	29	NA

Abbreviations: CT, computed tomography; ED, emergency department; GCS, Glasgow Coma Scale; hosp, hospitalization; ICU, intensive care unit; LOC, loss of consciousness; meds, medications; NA, not available; PTA, posttraumatic amnesia; PTSD, posttraumatic stress disorder; TBI, traumatic brain injury.

^a^ Statistical significance achieved by Mann-Whitney *U* test for all continuous or ordinal variables (except age group) and Fisher exact test for all categorical variables.

^b^ Other race/ethnicity categories included Indian, Alaska Native/Inuit, and Native Hawaiian/Pacific Islander.

^c^ Percentages of patients with unknown data have not been calculated and are not included because they are not relevant to the hypotheses tested.

In the primary analysis (Table 2), outcome measured by the cognitive, emotional, and somatic symptom clusters of the RPQ over the 4 assessment times was modeled using mixed-effects linear regression with random intercepts and slopes for each participant and an unstructured correlation matrix, with a specific interest in the interaction of injury group and sex. All models were adjusted for time (2 weeks, 3 months, 6 months, and 12 months), years of education, and type of insurance provider (Table 1) as a surrogate for socioeconomic status. All possible 2-way and 3-way interactions among time, injury group, and sex were initially included as covariates. Nonsignificant interactions (*P* > .05) were removed using backward elimination.^21^ If all interactions involving a variable were eliminated, that variable was then considered for elimination. If the primary interaction of injury group and sex was removed during this process, it was forced back into the model.

**Table 2.** TBI × Sex Interaction Models^a^

Variable	Rivermead cognitive		Rivermead emotional		Rivermead somatic		BSI depression		BSI anxiety		PHQ-9		PCL-5	
	B	*P* value	B	*P* value	B	*P* value	B	*P* value	B	*P* value	B	*P* value	B	*P* value
Intercept	1.76	<.001	3.11	<.001	2.90	<.001	4.23	<.001	3.69	<.001	7.48	<.001	21.74	<.001
Time	NA	<.001	NA	<.001	NA	<.001	NA	<.001	NA	.64	NA	<.001	NA	<.001
3 mo (vs 2 wk)	0.04	.82	−0.38	<.001	−0.66	<.001	−0.54	<.001	−0.20	.48	−1.59	<.001	−0.94	.32
6 mo (vs 2 wk)	0.30	.06	−0.49	<.001	−0.57	.001	−0.56	<.001	0.15	.60	−2.00	<.001	0.30	.76
12 mo (vs 2 wk)	0.53	.001	−0.56	<.001	−0.26	.12	−0.71	<.001	0.12	.68	−1.82	<.001	−0.37	.71
TBI	2.16	<.001	0.92	<.001	2.02	<.001	0.82	.01	1.86	<.001	1.74	<.001	7.67	<.001
Female sex	0.28	.35	0.64	.04	0.54	.09	0.70	.17	1.44	.007	0.63	.33	4.87	.01
Education years	−0.07	<.001	−0.10	<.001	−0.08	<.001	−0.15	<.001	−0.16	<.001	−0.18	<.001	−0.86	<.001
Insurance	NA	<.001	NA	<.001	NA	<.001	NA	<.001	NA	<.001	NA	<.001	NA	<.001
Insured/Medicare (vs none)	0.58	<.001	0.76	<.001	0.71	<.001	1.09	<.001	1.07	<.001	1.72	<.001	3.86	<.001
Medicaid/other (vs none)	0.07	.59	0.41	.005	0.30	.04	0.50	.04	0.56	.02	0.30	.32	2.72	.003
Time × TBI	NA	<.001	NA	NA	NA	<.001	NA	NA	NA	.002	NA	.05	NA	.004
3 mo (vs 2 wk)	−0.56	.001	NA	NA	−0.64	<.001	NA	NA	−0.55	.06	−0.49	.16	−2.10	.04
6 mo (vs 2 wk)	−0.68	<.001	NA	NA	−0.91	<.001	NA	NA	−0.90	.003	−0.32	.38	−3.14	.003
12 mo (vs 2 wk)	−1.01	<.001	NA	NA	−1.26	<.001	NA	NA	−1.12	<.001	−1.01	.006	−3.51	.001
Time × sex	NA	.005	NA	<.001	NA	<.001	NA	NA	NA	<.001	NA	NA	NA	NA
3 mo (vs 2 wk)	−0.20	.08	−0.26	.03	−0.29	.01	NA	NA	−0.60	.004	NA	NA	NA	NA
6 mo (vs 2 wk)	−0.36	.002	−0.49	<.001	−0.33	.006	NA	NA	−0.63	.003	NA	NA	NA	NA
12 mo (vs 2 wk)	−0.37	.002	−0.65	<.001	−0.53	<.001	NA	NA	−0.89	<.001	NA	NA	NA	NA
TBI × sex	0.76	.01	0.43	.20	0.80	.01	0.02	.97	0.39	.48	0.72	.29	−0.12	.95
TBI × sex (FDR-corrected)^b^	NA	.02	NA	.20	NA	.02	NA	NA	NA	NA	NA	NA	NA	NA

Abbreviations: BSI, Brief Symptom Inventory; FDR, false discovery rate; GCS, Glasgow Coma Scale; NA, not available; PCL-5, Posttraumatic Stress Disorder Checklist (Fifth Edition); PHQ-9, Patient Health Questionnaire-9; TBI, traumatic brain injury.

^a^ Statistical significance by propensity-weighted mixed-effects linear regression, using multiple imputation to address missingness in education and insurance.

Propensity weighting determined by a boosted regression model containing 25 effects (including education, GCS score, psychiatric history, and insurance type). Similar results were obtained without propensity weighting and when adding psychiatric history or cause of injury as additional covariates (eTable 1 in the Supplement). Interaction effects were selected via backward elimination, with TBI × sex added back into the model at the end if necessary. The 3-way interaction of time × TBI × sex was not statistically significant in any of the outcome models.

^b^ Adjustments for multiple comparisons were applied to the TBI × sex *P* values in just the primary Rivermead outcomes per Benjamini-Hochberg (*m* = 3, where *m* is the number of hypotheses tested) using an FDR of 5%.

All regression models were adjusted for missing outcome patterns using propensity weighting.^22^ A propensity weight for each time point was derived using a boosted logistic regression model predicting completion vs noncompletion of outcome based on demographic, injury, and history variables. The resulting propensity weights were proportional to the inverse of the probability of measure completion, then normalized so that the sum equaled the number of cases with the outcome measure present. Additionally, multiple imputation was used to address missingness primarily in the education and insurance variables in order to maximize power. All reported *P* values were 2-sided and unadjusted for multiple comparisons, with the exception of those for the interaction of time and injury group for the 3 Rivermead domains, which were adjusted for a false discovery rate of 5% per Benjamini-Hochberg (*m* = 3, where *m* is the total number of hypotheses tested).^23^

Secondary analyses were conducted on the other measures using similar methods to the primary analyses. As an exploratory analysis, we also tested the interaction of injury group and age among women over time (Table 3), with no adjustment for multiple comparisons. As noted previously, we stratified age as 18 to 34 years, 35 to 49 years, and older than 50 years based primarily on established trends for gradually reduced circulating female sex hormones from age 35 to 50 years. Sensitivity analyses included psychiatric history and cause of injury as additional covariates; they were excluded from the main analyses because of small numbers in some cells but did not change the main results (eTable 1 in the Supplement). All data were analyzed using SPSS software, version 26 (IBM Corporation) except for propensity modeling, which was run using the Toolkit for Weighting and Analysis of Nonequivalent Groups (twang) package (Rand Corporation) from August 19, 2020, to March 3, 2021.

**Table 3.** TBI × Age Interaction Models (Women Only)^a^

Variable	Primary models						Sensitivity analyses					
	Rivermead cognitive		Rivermead emotional		Rivermead somatic		Rivermead cognitive		Rivermead emotional		Rivermead somatic	
	B	*P* value	B	*P* value	B	*P* value	B	*P* value	B	*P* value	B	*P* value
Intercept	2.43	<.001	4.07	<.001	3.60	<.001	2.04	.002	3.79	<.001	3.25	<.001
Time	NA	.41	NA	<.001	NA	<.001	NA	.37	NA	<.001	NA	<.001
3 mo (vs 2 wk)	−0.62	.04	−0.64	.001	−1.00	<.001	−0.65	.02	−0.65	.001	−1.03	<.001
6 mo (vs 2 wk)	−0.36	.22	−0.97	<.001	−1.03	<.001	−0.34	.24	−0.97	<.001	−1.03	<.001
12 mo (vs 2 wk)	−0.10	.73	−1.20	<.001	−0.79	.004	−0.12	.69	−1.20	<.001	−0.82	.003
TBI	2.71	<.001	1.25	.004	2.40	<.001	2.77	<.001	1.26	.004	2.42	<.001
Education years	−0.10	.003	−0.14	<.001	−0.11	.002	−0.10	.002	−0.14	<.001	−0.10	.002
Insurance	NA	.03	NA	.007	NA	.003	NA	.03	NA	.004	NA	.001
Insured or Medicare (vs none)	0.65	.008	0.80	.003	0.80	.001	0.64	.008	0.81	.002	0.82	.001
Medicaid (vs none)	0.15	.57	0.46	.10	0.56	.04	0.26	.32	0.74	.03	0.64	.02
Psych history	NA	NA	NA	NA	NA	NA	NA	<.001	NA	<.001	NA	<.001
None (vs hospital)	NA	NA	NA	NA	NA	NA	0.84	<.001	0.91	<.001	0.78	.001
Help (vs hospital)	NA	NA	NA	NA	NA	NA	1.13	<.001	1.12	<.001	0.77	.001
Medication (vs hospital)	NA	NA	NA	NA	NA	NA	1.12	.007	1.29	.004	0.94	.03
Cause of Injury	NA	NA	NA	NA	NA	NA	NA	.14	NA	.06	NA	.16
Road traffic (vs fall)	NA	NA	NA	NA	NA	NA	0.39	.06	0.52	.02	0.36	.10
Other (vs fall)	NA	NA	NA	NA	NA	NA	0.43	.15	0.16	.62	0.51	.11
Age, y	NA	.75	NA	.72	NA	.69	NA	.43	NA	.44	NA	.40
35-49 (vs 17-34)	−0.25	.70	0.21	.77	0.36	.60	−0.04	.95	0.43	.53	0.50	.44
<50 (vs 17-34)	−0.23	.68	0.47	.42	0.46	.40	0.03	.96	0.74	.20	0.74	.19
<50 (vs 35-49)	0.02	.97	0.26	.71	0.11	.87	0.07	.91	0.31	.66	0.23	.77
Time × TBI	NA	.001	NA	NA	NA	<.001	NA	<.001	NA	NA	NA	.001
Time × age	NA	<.001	NA	NA	NA	NA	NA	<.001	NA	NA	NA	NA
TBI × age	NA	.21	NA	.07	NA	.04	NA	.27	NA	.08	NA	.04
TBI × 35-49 (vs 17-34)	1.20	.09	1.22	.11	1.65	.02	0.96	.16	1.00	.18	1.53	.03
TBI × <50 (vs 17-34)	0.12	.84	−0.50	.42	−0.01	.99	−0.09	.87	−0.69	.26	−0.20	.73
TBI × ≥50 (vs 35-49)	−1.07	.12	−1.73	.11	−1.66	.02	−1.05	.13	−1.69	.02	−1.56	.02

Abbreviations: NA, not available; TBI, traumatic brain injury.

^a^ Statistical significance by propensity-weighted mixed-effects linear regression, using multiple imputation to address missingness in education and insurance.

Propensity weighting determined by a boosted regression model containing 25 effects (including education, GCS score, psychiatric history, and insurance type). Interaction effects were selected via backward elimination, with TBI × age added back into the model at the end if necessary.

## Results

### Participant Characteristics

A total of 2000 patients with mTBI (1331 men [67%; mean (SD) age, 41.0 (17.3) years; 1026 White (78%)] and 669 women [33%; mean (SD) age, 43.0 (18.5) years; 505 White (76%)]) were evaluated. A total of 1133 patients (84%) had a GCS^24^ score of 13 to 15 (defined as mTBI), 717 (37%) had a positive CT finding (defined as complicated mTBI) and 1219 (63%) had a normal CT finding (defined as uncomplicated mTBI). There were 64 subjects for whom no CT information was available. The TBI group included 1331 men (67%), and the OTC group had 199 men (67%). The mTBI sample size with available outcome data ranged from 1508 (75%) at 2 weeks to 1262 (63%) at 12 months.

Within the mTBI group, men were significantly younger (mean [SD] age, 41.0 [17.3] years vs women, 43.0 [18.5] years; *P* = .07); had less education (mean [SD], 13.4 [2.9] years vs 13.7 [2.9] years; *P* = .001); were less likely to be insured (296 of 1254 [24%] vs 94 of 638 [15%]; *P* < .001); had higher prevalence of previous TBI (emergency visit, 170 [14%] vs 73 [12%]; *P* = .001); more frequently had a history of drug use (372 [30%] vs 130 [20%]; *P* < .001), a violent cause of injury (110 [8%] vs 18 [3%]; *P* = .001), and a positive CT (506 [39%] vs 211 [33%]; *P* = .004); exhibited longer durations of loss of consciousness (>24 hours, 6 [1%] vs 0; *P* = .006); and had a greater rate of intensive care unit admission (450 [34%] vs 180 [27%]; *P* = .001) (Table 1). Women were more likely to live independently (526 of 669 [87%] vs men, 1011 of 1331 [80%]; *P* = .001), pursue litigation (113 [25%] vs 161 [19%]; *P* = .04), and have a history of psychiatric issues including diagnoses of anxiety (130 [19%] vs 113 [9%]; *P* < .001), depression (151 [23%] vs 134 [10%]; *P* < .001), and sleep disturbance (34 [5%] vs 39 [3%]; *P* < .001). No significant sex differences were observed for race, Hispanic ethnicity, or presence of concomitant extracranial injury (Table 1).

Within the OTC group, men were significantly younger (mean [SD] age, 38.9 [14.6] years vs women, 43.3 [16.6] years; *P* = .03), had less education (mean [SD], 13.3 [3.1] years vs 14.7 [2.4] years; *P* < .001), were less insured (116 of 199 [62%] vs 68 of 100 [72%]; *P* = .03), and more often had a history of drug use (60 [31%] vs 20 [21%]; *P* = .02). Women were significantly more likely to live independently (89 [92%] vs 152 [80%] men; *P* = .002), have a history of depression (26 [26%] vs 20 [10%]; *P* < .001), and be injured from a fall (50 [52%] vs 53 [28%]; *P* < .001). No significant sex differences were observed for race, Hispanic ethnicity, highest level of care, or litigation (Table 1).

### Primary Outcome Assessments

Table 2 presents the results of models comparing injury group (TBI), sex, and time on primary and secondary outcomes, controlling for years of education and insurance type. Sensitivity analyses additionally adjusting for psychiatric history and cause of injury confirmed the major findings (eTable 1 in the Supplement). The TBI × sex × time interaction was not significant for any of the 3 RPQ symptom clusters.

#### Cognitive, Somatic, and Emotional Symptoms

**Figure 1.**
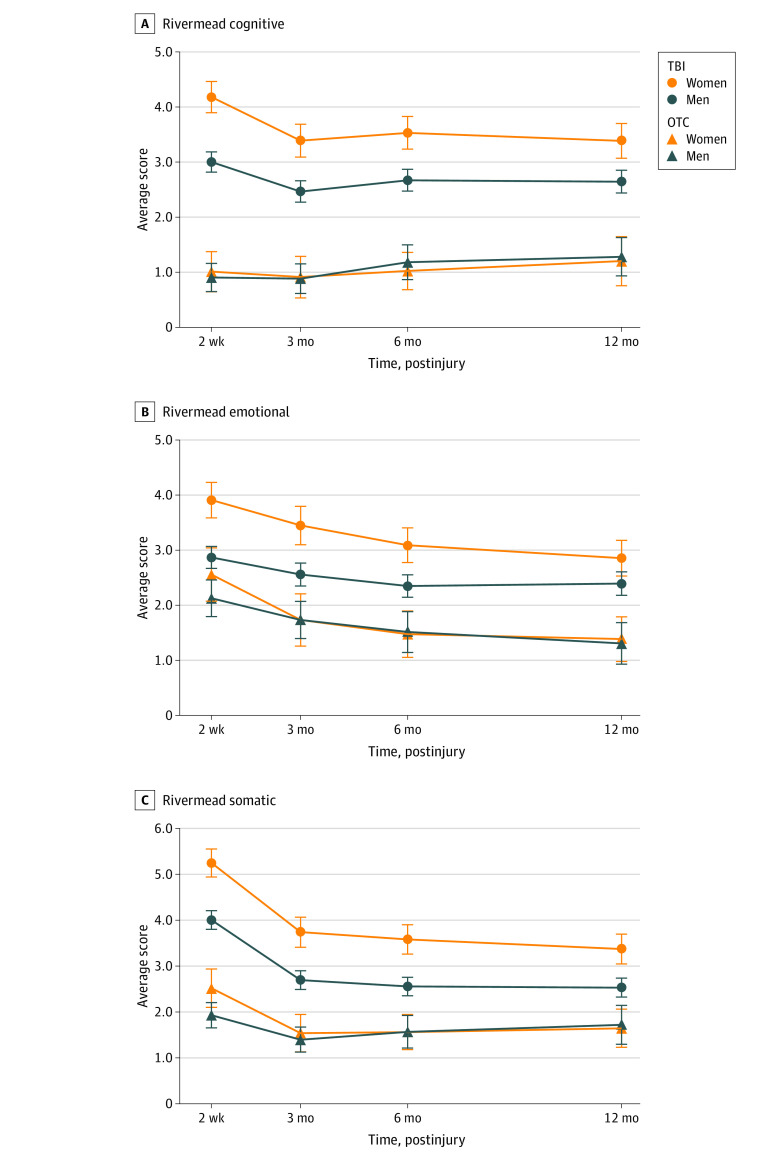
Postconcussion Symptoms Questionnaire (RPQ) Cognitive (A), Emotional (B), and Somatic (C) Cluster Scores Plotted by End Points for Women and Men with Traumatic Brain Injury (TBI) and Controls With Orthopedic Trauma (OTC)

The TBI × sex interaction was significant after adjustment for multiple comparisons for primary outcomes, including cognitive symptoms (B = 0.76; 5% false discovery rate–corrected *P* = .02) and somatic symptoms (B = 0.80; 5% false discovery rate–corrected *P* = .02) (Figure 1 and Table 2). Time × TBI was also significant, reflecting reduction in TBI vs OTC group differences over time as TBI participants recovered (eg, TBI vs OTC mean difference = 2.40 at 2 weeks and 1.54 at 12 months; *P* < .001) (eFigures 2 and 5 in the Supplement). The time × sex interaction was significant for emotional symptoms (Table 2), owing to the women-to-men difference in symptom severity diminishing over time (eg, mean difference 0.94 at 2 weeks vs 0.34 at 12 months; *P* < .001) (eFigures 2 and 5 in the Supplement).

The TBI × sex interaction (B = 0.80; *P* = .02) indicating more severe symptoms in female than male patients was significant after adjustment for multiple comparisons for primary outcomes. Time × sex interaction was significant (*P* < .001), suggesting that the difference in symptom severity between men and women decreased over time as TBI participants recovered from injury symptoms (men vs women mean difference = 1.21 at 2 weeks vs 0.69 at 12 months) (eTable 3 and eFigures 2 and 5 in the Supplement). Similarly, the time × TBI interaction was significant (*P* < .001), suggesting that the difference in symptom severity between TBI and OTC groups decreased over time as TBI participants recovered from injury symptoms (TBI vs OTC mean difference = 2.32 at 2 weeks vs 1.08 at 12 months) (eTable 3 and eFigure 5 in the Supplement).

### Other Outcome Measures

#### Posttraumatic Stress Disorder Checklist–5

Although we found no significant TBI × sex interaction (Table 2), the TBI × time interaction was significant (*P* = .004), indicating that the difference in PTSD symptoms between TBI and OTC decreased from 2 weeks (mean difference = 7.6) to 12 months (mean difference = 4.5) postinjury (eTable 3 and eFigures 2 and 5 in the Supplement). Road traffic accidents (B = 3.12 vs falls; 95% CI, 1.64-4.60; *P* < .001), other injury causes including violence (B = 3.76 vs falls; 95% CI, 1.81-5.71; *P* < .001), and lower education (B = –0.92 per education year; 95% CI, –1.16 to 0.68; *P* < .001) were also associated with greater PTSD symptom severity (eTable 1 in the Supplement).

#### Patient Health Questionnaire-9 and BSI-18 Anxiety and Depression

For the PHQ-9, the interaction of TBI × sex was not significant. Lower educational level was associated with greater PHQ-9 depression symptom severity (B = −0.21 per education year; *P* < .001) (eTable 1 and eFigure 3 in the Supplement).

Although the TBI × sex interactions were not significant for the BSI-anxiety and BSI-depression scales, there were significant time × sex (*P* < .001) and time × TBI (*P* = .002) interactions for BSI-anxiety. These interactions reflected that the magnitude of group differences decreased over time between women and men (mean difference = 1.73 at 2 weeks, 0.76 at 12 months) and between TBI and OTC groups (mean difference = 1.94 at 2 weeks, 0.91 at 12 months) (eTable 3 and eFigures 2 and 5 in the Supplement). Road traffic accidents (B = 0.51 vs falls; 95% CI, 0.12-0.91; *P* = .01) and other injury causes, including violence (B = 0.70 vs falls; 95% CI, 0.18-1.22; *P* = .008), were also associated with higher BSI-anxiety (eTable 1 and eFigure 4B in the Supplement). There were no significant interactions for BSI-18 depression.

### Analysis of Female Age Subgroups for the RPQ

**Figure 2.**
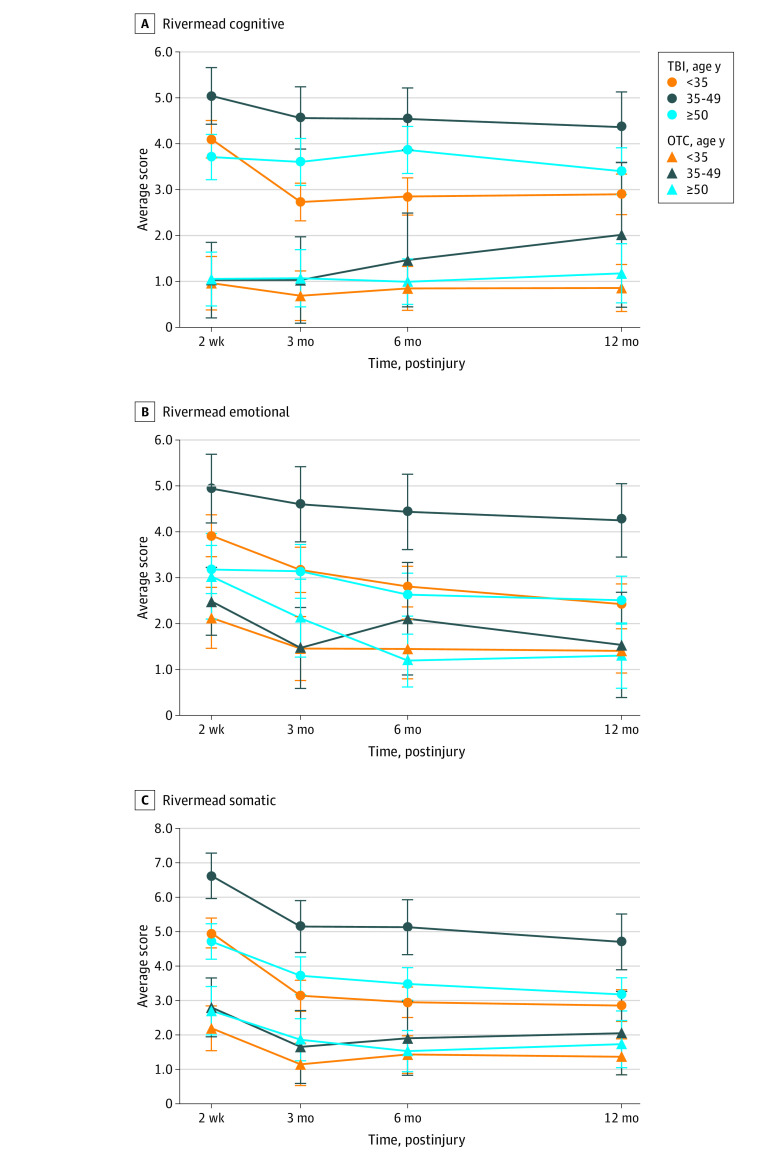
Postconcussion Symptoms Questionnaire (RPQ) Cluster Scores for Cognitive (A), Emotional (B), and Somatic (C) Clusters Plotted by End Points for Subgroups of Women With Traumatic Brain Injury and Controls With Orthopedic Trauma Aged 17-34 Years, 35-49 Years, and Older Than 50 Years

Given findings supporting the hypothesis that women were at greater risk of PCS than men, alongside the aforementioned work indicating potential age differences in recovery for women, we analyzed the outcome data separately for the female mTBI and OTC patients in 3 age strata: 17 to 34 years, 35 to 49 years, and older than 50 years (Table 3). Figure 2 illustrates the mean RPQ domain score by injury group and age group.

In an exploratory analysis looking at the different age groups, we found that the difference in symptom severity between mTBI and OTC groups was significantly greater in the age 35- to 49-year subgroup than the age 17- to 34-year subgroup for somatic (B = 1.65; 95% CI, 0.25-3.06; *P* = .02), but not cognitive or emotional symptoms. Similarly, the age 35- to 49-year subgroup had more severe somatic symptoms than the older than 50 years subgroup (B = 1.66; 95% CI, 0.25-3.07; *P* = .02). There were no differences between the older than 50 years and 17- to 34-year subgroups in severity of symptoms on any of the RPQ clusters. Time × TBI was significant, with symptom severity between mTBI and OTC groups improving from 2 weeks postinjury for cognitive symptoms (B = –0.64 at 3 months, B = –0.87 at 6 months, B = –1.20 at 12 months, *P* = .001), and somatic symptoms (B = –0.58 at 3 months, B = –0.76 at 6 months, B = –1.26 at 12 months, *P* < .001), but not emotional symptoms. In an exploratory analysis looking at the different age groups, we found that the difference in symptom severity between mTBI and OTC groups was significantly greater in the age 35- to 49-year subgroup than the age 17- to 34-year subgroup for somatic (B = 1.65; 95% CI, 0.25-3.06; *P* = .02), but not cognitive or emotional symptoms (95% CI, 0.25-3.07; *P* = .02).

## Discussion

In this cohort study using data from the TRACK-TBI study, we found that self-reported, TBI-related symptoms serially measured by the RPQ cognitive and RPQ somatic clusters over the first 12 months after injury were more severe in women than men with mTBI. Results suggest that women are increasingly at risk for TBI. Participation of women in contact sports and military combat is expanding, and physically active older women are vulnerable to falls.^2^ Meta-analyses have generally concluded that women are at greater risk for prolonged recovery from mTBI.^2,3^ In sports played by both female and male athletes, women are at greater risk than men for sustaining sports-related concussions.^3^ However, most studies of sex differences in mTBI outcome lack an extracranial injury control group to identify TBI-specific differences in recovery. The present study was designed to mitigate this confound by including an orthopedic injury comparison group. The finding of sex differences in RPQ symptoms in the mTBI but not the OTC group suggests that the higher propensity of women to experience prolonged mTBI recovery may not be a consequence of sex differences in preinjury symptoms or reporting biases. Furthermore, these difference may not be the result of the nonspecific effects of trauma; rather, the sex differences may be a direct result of differences in susceptibility to brain injury symptoms. Notably, our finding of more severe symptoms reported by female than male patients with mTBI on the RPQ emerged despite higher GCS scores indicating milder acute injury, lower rates of CT pathology and prior TBI, and a higher average educational level in women; this would have been expected to work against their hypothesized vulnerability to TBI-related symptoms.

We did not find significant interactions of TBI with sex for the other outcome measures (PCL-5, PHQ-9, BSI-18). In comparison with the RPQ, these measures do not compare the current severity of a symptom to preinjury level; therefore, changes after TBI are not as clearly defined. This dissociation has implications for structuring symptom measures in future TBI research.

In the analysis confined to female patients, we found that women aged 35 to 49 years had more severe somatic PCS after mTBI than women aged 17 to 34 years or older than 50 years. Although this finding is preliminary and should be interpreted accordingly, further investigation measuring psychosocial stress and sex hormone levels could examine the mechanisms underpinning this mTBI × age interaction. Greater psychosocial stress in women aged 35 to 49 years than in younger and older women is compatible with resuming multiple roles (eg, child care, family, and occupation)^25^; psychosocial stress has been postulated to explain the vulnerability of age-specific age subgroups^5,8^ to persistent PCS in women.^25^ Although the median age at onset of menopause is 51 years,^12^ levels of estrogen and progesterone begin to decline in the premenopausal age range, which begins at 35 to 40 years.^13,14^ Whether declining levels of these hormones is contributory to the present findings awaits further investigation.

Our finding of the vulnerability of women in the 35- to 49-year age range to chronic mTBI effects was also compatible with the results of a recent multicenter study that showed that the greater difference in women vs men with PCS after mTBI was in the 16- to 45-year age range, but we acknowledge the difference in age grouping between the studies.^8^ As noted previously, psychosocial stress may contribute to greater PCS in the women aged 35 to 49 years than in younger females after TBI.^26^

The contribution of psychosocial stress was also proposed by Yue et al^5^ to account for the more severe PTSD symptoms reported by women aged 30 to 39 years with mTBI as compared with women aged 18 to 29 years and men of either age range in a TRACK-TBI pilot study. Definitive testing of this hypothesized interaction would require measurement of circulating sex hormones to characterize the menstrual cycle of individual women in the reproductive age range. Measurement of daily stress, including self-report, validated indicators, and objective biomarkers such as cytokines or cortisol would also be necessary to investigate this interaction. Additionally, retrospective assessment of environmental stressors during the preinjury period and serial measurement during postinjury follow-up would inform the role of stress and its interaction with circulating sex hormones.

### Strengths and Limitations

Strengths of this study include enrollment of the orthopedic injury group, which controlled for the nonspecific effects of traumatic injury and sex on symptom reporting. Our finding that sex differences in self-reported symptoms on the RPQ cognitive and somatic symptom clusters were specific to the mTBI group mitigates concern that this vulnerability among women may be characteristic of acute traumatic injury in general and implicates brain mechanisms.

We acknowledge imbalances between the men and women with TBI groups, including education, prior TBI, preinjury psychiatric disorder, cause of injury, health insurance, severity and duration of acute impairment of consciousness, and pathology identified by CT. Although the statistical analysis adjusted for some of these factors, including education, insurance, and cause of injury (motor vehicle crash vs other causes), other factors that we did not control for were in the opposite direction of our hypothesis and findings. Covarying for preinjury psychiatric disorder did not alter the results. The focus of this study and of the TRACK-TBI pilot study on cohorts enrolled at level 1 trauma centers and restricted to TBI patients triaged to CT within 24 hours after injury biased against patients with mTBI whose acute clinical status in the emergency department was less severe. Measurement of estrogen and progesterone levels and obtaining menstrual diaries were beyond the scope of this study, precluding objective evaluation of whether alterations of sex hormones contributed to persistent PCS. Similarly, perceived stress in daily life was not measured.

## Conclusions

Implications of this cohort study for individualized clinical management of mTBI include educating female patients about the greater risk of persistent symptoms and lengthier recovery after mTBI. Our findings suggest that female sex is a risk factor of which clinicians should be aware when triaging patients for follow-up. Although we found preliminary evidence that the risk of persistent PCS in women was greatest in the group aged 35 to 49 years, further study is needed to corroborate this finding and identify the mechanism.

## Notes

Supplement. **eFigure 1.** Patient Consort Diagram **eFigure 2.** PCL-5 Scores Plotted by End Points for Female and Male TBI and OTC Groups **eFigure 3.** PHQ-9 Scores Plotted by End Points for Female and Male TBI and OTC Groups **eFigure 4.** BSI-18 Cluster Scores for Depression (A), and Anxiety (B) Clusters Plotted by End Points for Female and Male TBI and OTC Groups **eFigure 5.** Average Scores for Rivermead Cognitive (A), Emotional (B), Somatic (C, D) and BSI Anxiety (E,F), and PCL (G) Plotted against Time Postinjury **eTable 1.** TBI × Sex Interaction Models **eTable 2.** Preinjury Demographics, Clinical History, and TBI Variables Stratified by Injury Type and Age Group **eTable 3.** Mean Outcome Scores by Injury Group and Sex
