# Supplementary material for: Association of Sex and Age With Mild Traumatic Brain Injury–Related Symptoms: A TRACK-TBI Study
Source: JAMA Netw Open. 2021 Apr 6;4(4):e213046. doi: 10.1001/jamanetworkopen.2021.3046 (PMC8025125; doi:10.1001/jamanetworkopen.2021.3046)
Supplement: Supplement. — eFigure 1. Patient Consort Diagram eFigure 2. PCL-5 Scores Plotted by End Points for Female and Male TBI and OTC Groups eFigure 3. PHQ-9 Scores Plotted by End Points for Female and Male TBI and OTC Groups eFigure 4. BSI-18 Cluster Scores for Depression (A), and Anxiety (B) Clusters Plotted by End Points for Female and Male TBI and OTC Groups eFigure 5. Average Scores for Rivermead Cognitive (A), Emotional (B), Somatic (C, D) and BSI Anxiety (E,F), and PCL (G) Plotted against Time Postinjury eTable 1. TBI × Sex Interaction Models eTable 2. Preinjury Demographics, Clinical History, and TBI Variables Stratified by Injury Type and Age Group eTable 3. Mean Outcome Scores by Injury Group and Sex [file jamanetwopen-e213046-s001.pdf]

Supplementary Online Content

Levin HS, Temkin NR, Barber J, et al; TRACK-TBI Investigators. Association of sex and age with mild traumatic brain injury–related symptoms: a TRACK-TBI study. *JAMA Netw Open*. 2021;4(4):e213046. doi:10.1001/jamanetworkopen.2021.3046

eFigure 1. Patient Consort Diagram

eFigure 2. PCL-5 Scores Plotted by End Points for Female and Male TBI and OTC Groups

eFigure 3. PHQ-9 Scores Plotted by End Points for Female and Male TBI and OTC Groups

eFigure 4. BSI-18 Cluster Scores for Depression (A), and Anxiety (B) Clusters Plotted by End Points for Female and Male TBI and OTC Groups

eFigure 5. Average Scores for Rivermead Cognitive (A), Emotional (B), Somatic (C, D) and BSI Anxiety (E,F), and PCL (G) Plotted against Time Postinjury

eTable 1. TBI × Sex Interaction Models

eTable 2. Preinjury Demographics, Clinical History, and TBI Variables Stratified by Injury Type and Age Group

eTable 3. Mean Outcome Scores by Injury Group and Sex

This supplementary material has been provided by the authors to give readers additional information about their work.

© 2021 Levin HS et al. *JAMA Network Open.*

eFigure 1. Patient Consort Diagram

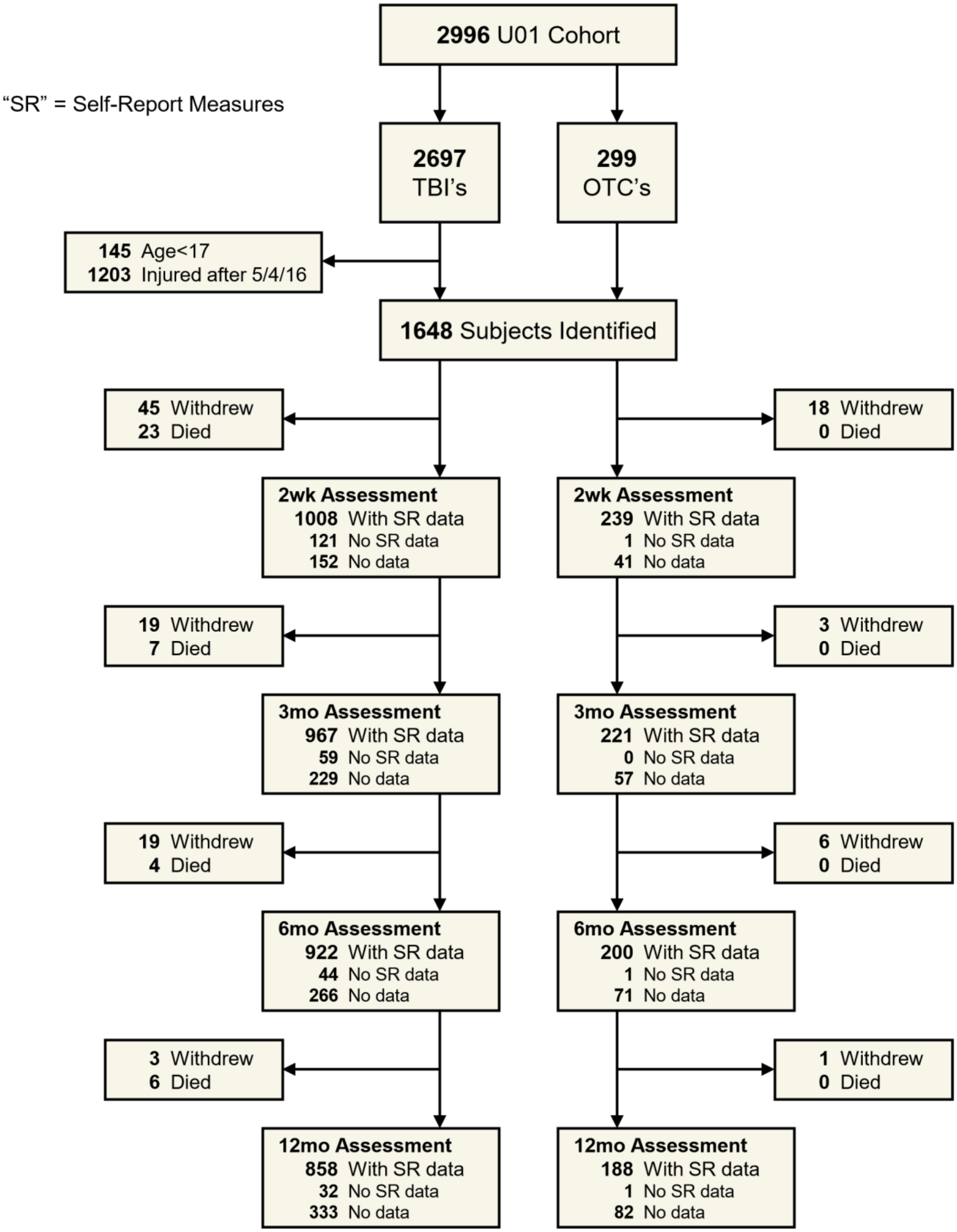

© 2021 Levin HS et al. *JAMA Network Open.*

eFigure 2. PCL-5 Scores Plotted by Endpoints for Female and Male TBI

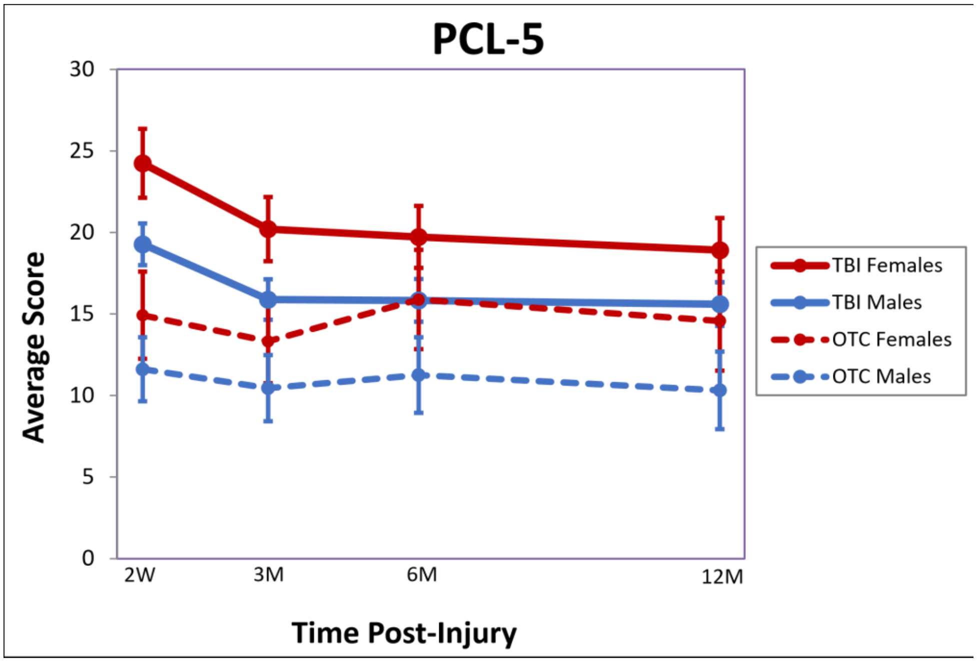

and OTC Groups

© 2021 Levin HS et al. *JAMA Network Open.*

eFigure 3. PHQ-9 Scores Plotted by Endpoints for Female and Male TBI and OTC Groups

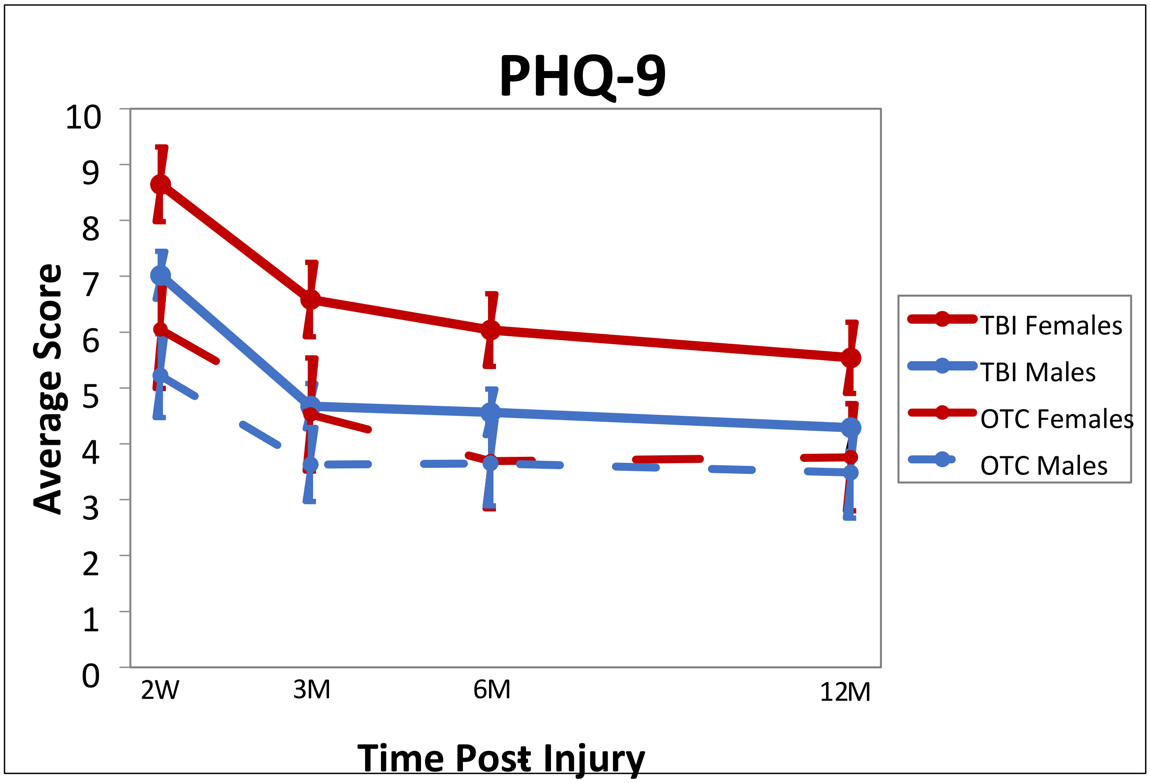

© 2021 Levin HS et al. *JAMA Network Open.*

eFigure 4. BSI-18 Cluster Scores for Depression (A), and Anxiety (B) Clusters Plotted by Endpoints for Female and Male TBI and OTC Groups

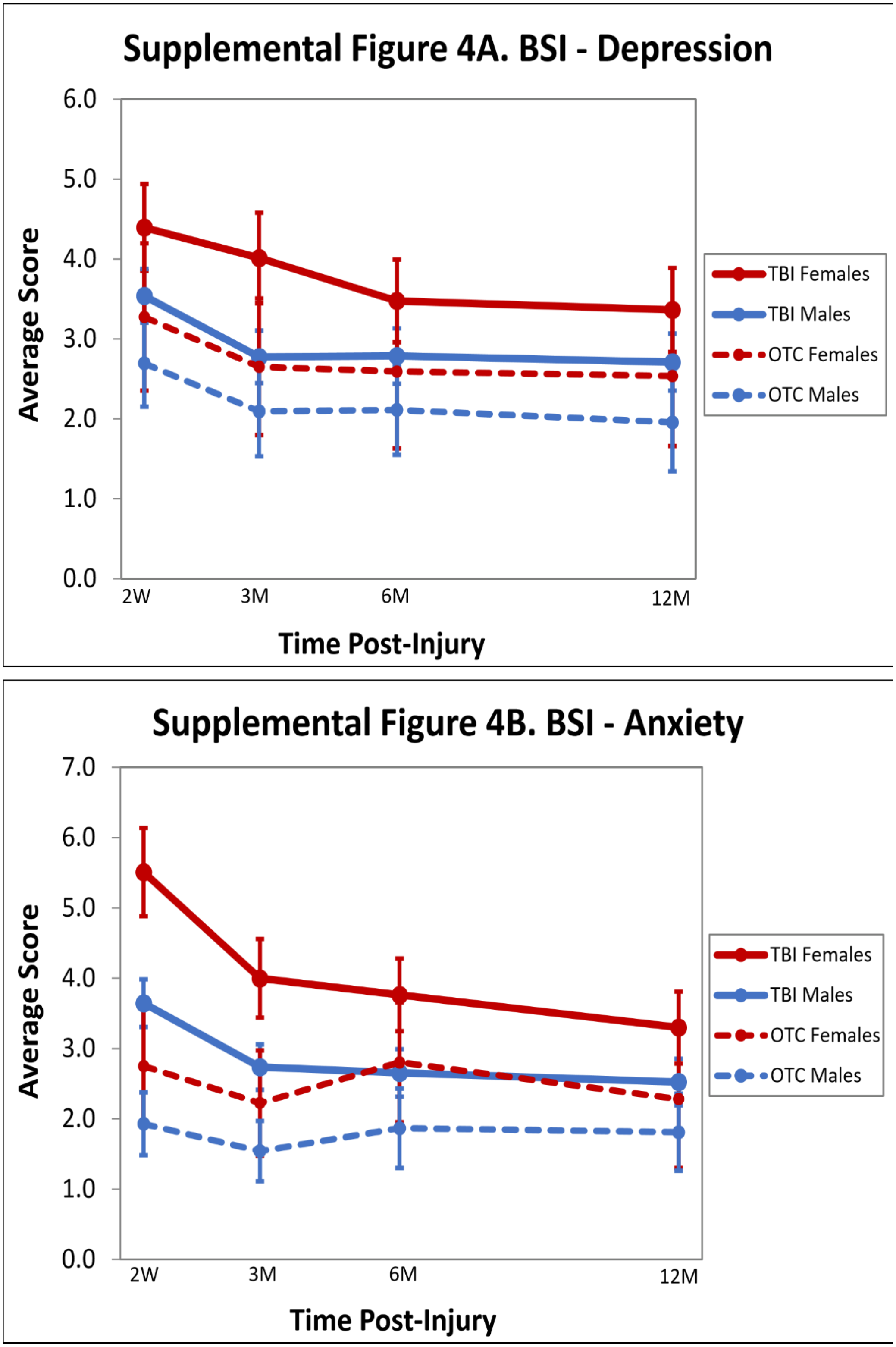

© 2021 Levin HS et al. *JAMA Network Open.*

eFigure 5. Average Scores for Rivermead Cognitive (A), Emotional (B), Somatic (C, D) and BSI Anxiety (E,F), and PCL (G) Plotted against Time Postinjury

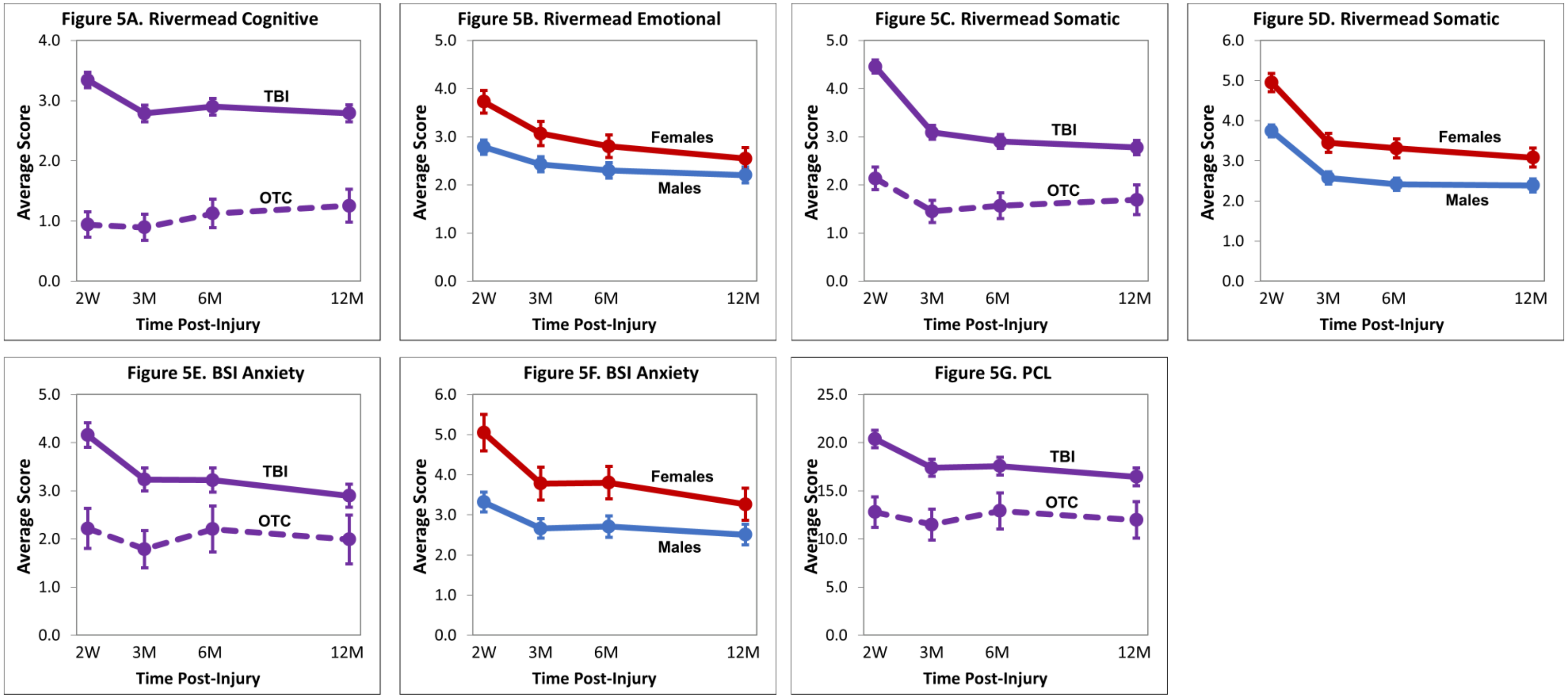

© 2021 Levin HS et al. *JAMA Network Open.*

eTable 1. TBI × Sex Interaction Models (plus Psych History and Injury Cause as sensitivity) (continued)

	Rivermead Cognitive		Rivermead Emotional		Rivermead Somatic		BSI Depression		BSI Anxiety		PHQ		PCL	
	β	p	β	p	β	p	β	p	β	p	β	p	β	p
Intercept	1.65	<.001	3.13	<.001	2.72	<.001	4.09	<.001	3.37	<.001	7.57	<.001	20.98	<.001
Time	---	.004	---	<.001	---	<.001	---	<.001	---	.56	---	<.001	---	.47
3mo (vs 2wk)	-0.01	.96	-0.38	<.001	-0.69	<.001	-0.53	<.001	-0.23	.43	-2.00	<.001	-1.23	.20
6mo (vs 2wk)	0.31	.06	-0.48	<.001	-0.56	.001	-0.55	<.001	0.17	.58	-2.26	<.001	0.21	.83
12mo (vs 2wk)	0.52	.002	-0.55	<.001	-0.26	.13	-0.70	<.001	0.12	.69	-2.72	<.001	-0.43	.67
TBI	2.17	<.001	0.94	<.001	2.08	<.001	0.87	.006	1.95	<.001	1.35	.001	7.79	<.001
Sex (Female)	0.15	.61	0.56	.08	0.50	.11	0.38	.45	1.35	.010	0.29	.65	4.51	.02
Education Years	-0.09	<.001	-0.11	<.001	-0.09	<.001	-0.17	<.001	-0.18	<.001	-0.21	<.001	-0.92	<.001
Insurance	---	.002	---	<.001	---	<.001	---	<.001	---	<.001	---	<.001	---	.001
Insured/Medicare (vs None)	0.51	.001	0.68	<.001	0.65	<.001	0.87	.001	0.86	.001	1.49	<.001	3.09	.002
Medicaid/ Other (vs None)	0.13	.33	0.45	.001	0.34	.02	0.63	.008	0.64	.007	0.45	.13	2.88	.001
Psych History		<.001		<.001		<.001		<.001		<.001		<.001		<.001
Help (vs None)	0.67	<.001	0.75	<.001	0.61	<.001	1.70	<.001	1.47	<.001	1.90	<.001	5.46	<.001
Meds (vs None)	1.19	<.001	1.23	<.001	0.97	<.001	2.36	<.001	2.02	<.001	3.13	<.001	7.65	<.001
Hospitalization (vs None)	1.62	<.001	1.75	<.001	1.48	<.001	5.12	<.001	4.55	<.001	5.26	<.001	15.80	<.001
Cause of Injury	---	.31	---	.004	---	.15	---	.64	---	.01	---	.14	---	<.001
Road Traffic (vs Fall)	0.15	.17	0.41	.001	0.16	.19	0.16	.43	0.51	.01	0.48	.05	3.12	<.001
Other (vs Fall)	0.19	.20	0.26	.10	0.30	.06	0.22	.40	0.20	.008	0.42	.20	3.76	<.001
Time * TBI	---	<.001	---	N/A	---	<.001	---	N/A	---	.002	---	N/A	---	.007
3mo (vs 2wk)	-0.51	.002	N/A	N/A	-0.60	<.001	N/A	N/A	-0.51	.09	N/A	N/A	-1.80	.08

© 2021 Levin HS et al. *JAMA Network Open.*

6mo (vs 2wk)	-0.69	<.001	N/A	N/A	-0.91	<.001	N/A	N/A	-0.91	.003	N/A	N/A	-3.03	.004
12mo (vs 2wk)	-1.00	<.001	N/A	N/A	-1.25	<.001	N/A	N/A	-1.10	<.001	N/A	N/A	-3.40	.002
Time * Sex	---	.007	---	<.001	---	<.001	---	N/A	---	<.001	---	N/A	---	N/A
3mo (vs 2wk)	-0.19	.09	-0.26	.03	-0.29	.02	N/A	N/A	-0.61	.004	N/A	N/A	N/A	N/A
6mo (vs 2wk)	-0.35	.003	-0.49	<.001	-0.33	.005	N/A	N/A	-0.65	.002	N/A	N/A	N/A	N/A
12mo (vs 2wk)	-0.36	.003	-0.65	<.001	-0.54	<.001	N/A	N/A	-0.89	<.001	N/A	N/A	N/A	N/A
TBI * Sex	0.74	.02	0.34	.30	0.72	.03	0.01	.99	0.21	.70	0.64	.34	-0.84	.68

Statistical significance by propensity-weighted mixed-effects regression, using multiple imputation to address missingness in education. Propensity weighting determined by a boosted regression model containing 25 effects (including education, GCS score, psychiatric history, and insurance type. Interaction effects were selected via backward elimination, with TBI*sex added back into the model at the end if necessary. Cells marked N/A indicate that the effect was not included in the model for that outcome.

© 2021 Levin HS et al. *JAMA Network Open.*

eTable 2. Preinjury Demographics, Clinical History, and TBI Variables Stratified by Injury Type and Age Group

	mTBI (Females Only)				Trauma-Controls (Females Only)			
	**Age 17-34 N=273 (41%)**	**Age 35-49 N=138 (21%)**	**Age 50+ N=258 (39%)**	**p**	**Age 17-34 N=39 (39%)**	**Age 35-49 N=21 (21%)**	**Age 50+ N=40 (40%)**	**p**
**Race**								
White	190 (70%)	102 (74%)	213 (84%)	.006	32 (82%)	13 (65%)	30 (75%)	.74
Black	58 (21%)	29 (21%)	32 (13%)		5 (13%)	6 (30%)	7 (18%)	
Asian	11 (4%)	4 (3%)	7 (3%)		1 (3%)	1 (5%)	1 (3%)	
Mixed Race	9 (3%)	2 (1%)	1 (0%)		1 (3%)	0 (0%)	1 (3%)	
Other	2 (1%)	1 (1%)	0 (0%)		0 (0%)	0 (0%)	1 (3%)	
Unknown	3	0	5		0	1	0	
**Hispanic**								
No	204 (76%)	111 (80%)	223 (88%)	.001	26 (67%)	18 (90%)	35 (88%)	.04
Yes	66 (24%)	27 (20%)	31 (12%)		13 (33%)	2 (10%)	5 (13%)	
Unknown	3	0	4		0	1	0	
**Education**								
Mean (SD)	13.1 (2.3)	13.7 (3.0)	13.8 (3.5)	<.001	13.7 (2.5)	13.5 (3.1)	14.0 (3.3)	.94
Unknown	43	20	39		3	3	1	
**Insurance**								
Insured/Medicare	138 (53%)	86 (65%)	202 (82%)	<.001	25 (68%)	12 (63%)	31 (79%)	.32
Medicaid/Other	67 (26%)	26 (20%)	25 (10%)		6 (16%)	6 (32%)	5 (13%)	
Uninsured	55 (21%)	21 (16%)	18 (7%)		6 (16%)	1 (5%)	3 (8%)	
Unknown	13	5	13		2	2	1	
**Living Situation** SesPrimAdult								
Independent Living (1-3)	191 (72%)	129 (95%)	242 (98%)	<.001	33 (87%)	19 (95%)	37 (95%)	.14
Dependent on Others (4,5)	70 (27%)	6 (4%)	6 (2%)		5 (13%)	0 (0%)	1 (3%)	
Other (6-9,10-15,99)	3 (1%)	1 (1%)	0 (0%)		0 (0%)	1 (5%)	1 (3%)	
Unknown	9	2	10		1	1	1	
**Previous TBI**								

© 2021 Levin HS et al. *JAMA Network Open.*

No	211 (83%)	99 (77%)	196 (84%)		31 (86%)	15 (83%)	32 (86%)	
ED Visit	32 (13%)	19 (15%)	22 (9%)	.21	4 (11%)	0 (0%)	2 (5%)	.90
Hospital Admit	10 (4%)	10 (8%)	14 (6%)		1 (3%)	3 (17%)	3 (8%)	
Unknown	20	10	26		3	3	3	
**Drug Use History**								
None	161 (61%)	108 (82%)	226 (93%)		23 (61%)	13 (68%)	38 (97%)	
Yes, no trouble	95 (36%)	20 (15%)	15 (6%)	<.001	14 (37%)	5 (26%)	1 (3%)	<.001
Yes, with trouble	6 (2%)	4 (3%)	2 (1%)		1 (3%)	1 (5%)	0 (0%)	
Unknown	11	6	15		1	2	1	
**Psych History**								
None	167 (61%)	70 (51%)	141 (55%)		17 (44%)	11 (52%)	22 (55%)	
Received help (no regular meds)	52 (19%)	30 (22%)	45 (17%)	.08	8 (21%)	6 (29%)	6 (15%)	.53
Used meds regularly (no hosp.)	39 (14%)	28 (20%)	69 (27%)		11 (28%)	2 (10%)	11 (28%)	
Hospitalized	15 (5%)	10 (7%)	3 (1%)		3 (8%)	2 (10%)	1 (3%)	
**Psych History**								
Anxiety	58 (21%)	30 (22%)	42 (16%)	.25	7 (18%)	5 (24%)	7 (18%)	.85
Depression	58 (21%)	35 (25%)	58 (22%)	.64	11 (28%)	3 (14%)	12 (30%)	.41
Sleep Disorders	11 (4%)	12 (9%)	11 (4%)	.12	1 (3%)	1 (5%)	1 (3%)	1.00
Bipolar Disorder	7 (3%)	4 (3%)	4 (2%)	.63	1 (3%)	0 (0%)	0 (0%)	.61
Schizophrenia	2 (1%)	0 (0%)	0 (0%)	.52	0 (0%)	1 (5%)	0 (0%)	.21
PTSD	4 (1%)	9 (7%)	2 (1%)	.003	0 (0%)	0 (0%)	0 (0%)	---
Other	9 (3%)	1 (1%)	7 (3%)	.29	1 (3%)	0 (0%)	1 (3%)	1.00
**Injury Cause**								
Road Traffic Accident	205 (75%)	87 (64%)	118 (46%)		17 (44%)	9 (43%)	9 (24%)	
Fall	32 (12%)	35 (26%)	117 (46%)		14 (36%)	9 (43%)	27 (73%)	
Other Accident	13 (5%)	6 (4%)	8 (3%)	<.001	4 (10%)	3 (14%)	1 (3%)	.01
Violence	8 (3%)	3 (2%)	7 (3%)		0 (0%)	0 (0%)	0 (0%)	
Other	15 (5%)	6 (4%)	6 (2%)		4 (10%)	0 (0%)	0 (0%)	

© 2021 Levin HS et al. *JAMA Network Open.*

Unknown	0	1	2		0	0	3	
**ER GCS**								
Mean (SD)	14.7 (0.6)	14.7 (0.5)	14.7 (0.5)	.11				
**CT Positive**								
No	220 (81%)	90 (66%)	128 (53%)	<.001				
Yes	50 (19%)	46 (34%)	115 (47%)					
Unknown	3	2	15					
**LOC Duration**								
None	19 (10%)	14 (15%)	41 (23%)	.003				
1-29 minutes	157 (84%)	74 (81%)	129 (73%)					
30min-24 hrs	11 (6%)	3 (3%)	6 (3%)					
>24 hours	0 (0%)	0 (0%)	0 (0%)					
Unknown	86	47	82					
**PTA Duration**								
None	33 (18%)	18 (18%)	41 (24%)	.48				
1-29 minutes	82 (44%)	50 (50%)	71 (41%)					
30min-24 hrs	57 (31%)	25 (25%)	52 (30%)					
>24 hours	13 (7%)	7 (7%)	8 (5%)					
Unknown	88	38	86					
**Highest Level of Care**								
ED	87 (32%)	44 (32%)	62 (24%)	.004	21 (54%)	7 (33%)	16 (40%)	.25
Ward	131 (48%)	56 (41%)	109 (42%)		15 (38%)	11 (52%)	20 (50%)	
ICU	55 (20%)	38 (28%)	87 (34%)		3 (8%)	3 (14%)	4 (10%)	
**Litigation at 12 Months**								
No	133 (76%)	59 (61%)	155 (83%)	<.001	25 (89%)	11 (85%)	24 (80%)	.63
Yes	43 (24%)	38 (39%)	32 (17%)		3 (11%)	2 (15%)	6 (20%)	
Unknown	97	41	71		11	8	10	

*TBI: Traumatic Brain Injury; ED: Emergency Department; PTSD: Post-Traumatic Stress Disorder; GCS: Glasgow Coma Scale;
CT: Computed Tomography; ICU=Intensive Care Unit; LOC: Loss of Consciousness; PTA: Post Traumatic Amnesia; ED: Emergency Department; ICU: Intensive Care Unit

Statistical significance by Mann-Whitney for all continuous/ordinal variables (except age group) and Fisher's exact test for all categorical variables

© 2021 Levin HS et al. *JAMA Network Open.*

eTable 3. Mean Outcome Scores by Injury Group and Sex

Measure	Time	Injury Group			Sex		
		TBI	OTC	Diff.	Female	Male	Diff.
Rivermead Cognitive	2Wk	3.34	0.94	2.40	3.67	2.70	0.97
	3Mo	2.79	0.90	1.89	3.01	2.30	0.71
	6Mo	2.90	1.13	1.77	3.05	2.47	0.58
	12Mo	2.79	1.25	1.54	2.97	2.39	0.58
Rivermead Emotional	2Wk	3.23	2.28	0.95	3.73	2.78	0.94
	3Mo	2.79	1.73	1.06	3.07	2.43	0.64
	6Mo	2.62	1.50	1.12	2.80	2.30	0.50
	12Mo	2.47	1.34	1.13	2.55	2.21	0.34
Rivermead Somatic	2Wk	4.46	2.14	2.32	4.95	3.75	1.21
	3Mo	3.09	1.45	1.64	3.45	2.57	0.88
	6Mo	2.90	1.57	1.34	3.31	2.42	0.90
	12Mo	2.78	1.69	1.08	3.08	2.39	0.69
BSI Depression	2Wk	3.72	2.90	0.82	4.17	3.33	0.84
	3Mo	3.26	2.30	0.96	3.74	2.81	0.93
	6Mo	3.22	2.28	0.93	3.45	2.91	0.54

© 2021 Levin HS et al. *JAMA Network Open.*

	12Mo	3.01	2.18	0.83	3.22	2.72	0.50
BSI Anxiety	2Wk	4.16	2.22	1.94	5.05	3.32	1.73
	3Mo	3.24	1.79	1.45	3.78	2.66	1.12
	6Mo	3.22	2.20	1.02	3.80	2.71	1.09
	12Mo	2.90	1.99	0.91	3.26	2.51	0.76
PHQ	2Wk	7.46	5.51	1.95	8.29	6.64	1.65
	3Mo	5.53	3.95	1.58	6.22	4.85	1.37
	6Mo	5.25	3.66	1.59	5.84	4.61	1.23
	12Mo	4.73	3.59	1.14	5.22	4.23	0.99
PCL	2Wk	12.8	20.4	7.6	17.6	22.9	5.3
	3Mo	11.5	17.4	5.9	15.2	19.2	4.0
	6Mo	12.9	17.6	4.7	15.5	19.8	4.3
	12Mo	12.0	16.5	4.5	14.6	18.2	3.6

© 2021 Levin HS et al. *JAMA Network Open.*
